# A study on signal enhancement of a Raman probe using an optical pickup unit

**DOI:** 10.1016/j.heliyon.2022.e10802

**Published:** 2022-09-29

**Authors:** Sung Il Ahn

**Affiliations:** Department of Chemistry Education, Graduate Department of Chemical Materials, Institute for Plastic Information and Energy Materials, Pusan National University, Busandaehakro 63-2, Busan 46241, Republic of Korea

**Keywords:** Raman spectrometer, Diode laser, Optical pickup unit, Tracking mode

## Abstract

A simplified fabrication method for an optical pickup unit (OPU)-based Raman probe was introduced. The use of OPU tracking mode was examined to increase the Raman signal and reduce background signals. Unlike most Raman systems, the tracking mode of the OPU moves the objective lens in the x (or y) direction, thus resulting in a significantly reduced background signal and increased Raman signal intensity. These results can be explained by two factors, such as reducing the surface scattering of the incident light from the objective lens and decreasing signal blocking by a dot mirror, which depends on the horizontal position of the objective lens. The reproducibility of the OPU Raman in tracking mode is higher than the OPU Raman in normal mode. The peak position deviation was calculated to be around ±3 cm^−1^. The Raman spectra of normal organic samples were obtained under the same conditions and recorded as per the applied voltage in the OPU tracking mode to validate the fabricated device. The objective lens shifts resulted in increased Raman peaks and reduced background signals. The maximum peak-position difference for all samples was 10 cm^−1^ compared to reference peaks obtained using a commercial Raman system.

## 1 Introduction

Raman spectroscopy has been used in many fields to analyze the structure of materials since it was discovered by C. V. Raman in 1928 [1]. The molecular structure of the scattering material, i.e., its inherent vibration energy, determines whether energy is absorbed or released. Based on this phenomenon, the molecular structure of a material can be estimated by analyzing its Raman scattered light. Unlike infrared (IR) spectroscopy, which measures the energy absorbed by the vibrations of a molecule having a change in dipole moment, Raman spectroscopy measures the energy lost or gained of scattered light compared with the incident light to measure the vibrational energies of a molecule having a change in polarizability. Furthermore, Raman spectroscopy does not restrict the size or shape of the studied samples; Raman analysis can be performed without pretreatment, and a minute amount of sample can be measured. Furthermore, the Raman signal of water molecules creates a minute signal, thus resulting in the easy measurement of water-soluble samples. Raman spectroscopy is a complementary analysis method for IR spectroscopy because it can detect vibration bands that are difficult to detect in IR spectra [2]. For the last two decades, low-dimensional carbon materials, such as graphene and carbon nanotubes, have attracted considerable attention from researchers. Although these materials emit very weak IR signals, Raman, especially resonance Raman spectroscopy, provides well-defined signals and has been extensively used for their analysis [3, 4, 5, 6, 7, 8, 9, 10]. Raman spectroscopy, in addition to IR and other spectroscopy techniques, is a vital analytical tool used in modern laboratories. Several educational experiments involving Raman spectroscopy have been reported [11, 12]. However, most commercially available Raman spectrometers for educational or research purposes are expensive. Several studies such as the development of low-cost Raman spectrometers have been conducted to address this issue [13, 14, 15, 16].

Alternatively, an optical pickup unit (OPU), which is used for optical disks, is a sophisticated, inexpensive, and small optical device. It is not only inexpensive but also advantageous in a spectrometer because of its precise optical path and small size. Many attempts have been made to use an OPU as a base and apply it to various types of optical devices. For example, previous studies have reviewed its feasibility for use with optical microscopes [17, 18] precise devices for determining refractive index at the femtoliter volume level [18, 19] and in biosensing (micro or nanoscale biological sensors) [20], high-speed contact-mode atomic force microscopy [21], and optical imaging modules for astigmatic detection systems [22].

**Figure 1:**
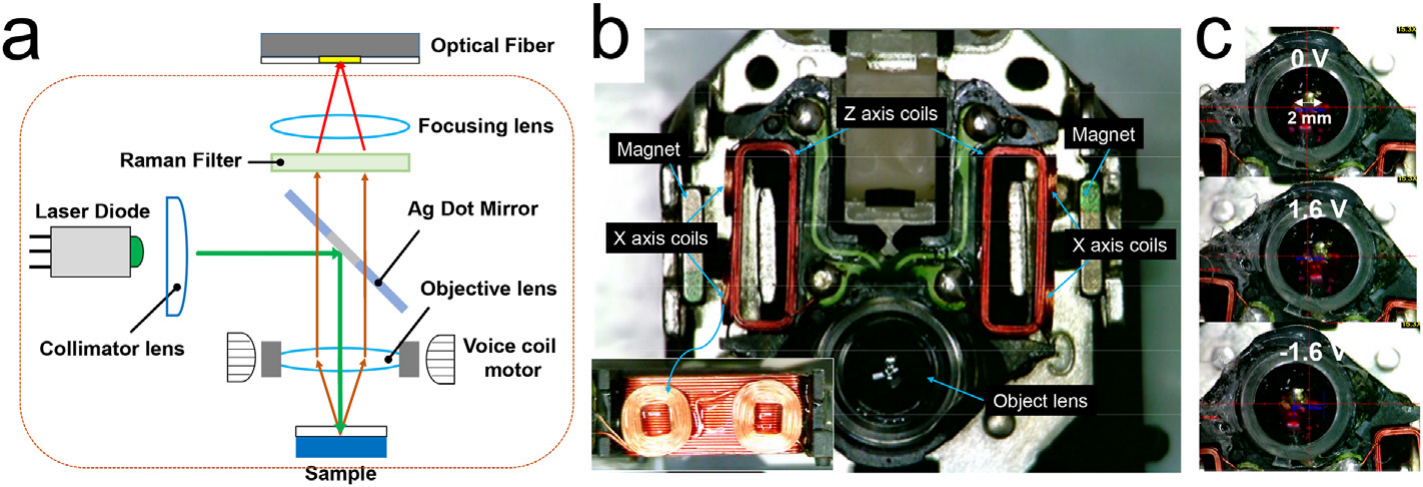
(a) Schematic of OPU-Raman probe, (b) Bottom structure of an OPU showing two magnetic coils (the inset is the side image of the x-axis coils), and (c) movement of the objective lens as per the tracking mode signal at 1.6 and −1.6 V.

In this study, a Raman probe was developed using an OPU because the structural characteristics of a Raman spectrometer are similar to those of an OPU (Figure 1a). An OPU-based Raman system has been reported in previous studies [23, 24]. However, the fabrication method discussed in previous studies is extremely complicated for educational purposes because of the replacement of multiple OPU parts with optical components. Moreover, the focus and tracking roles of the electromagnetic coil in the OPU were not considered. The OPU has a coil that moves the objective lens in the x (or y) direction to trace the laser path such that an optical disk can be correctly read (Figure 1b). The moving range of the lens is ∼0.8 mm in both directions (Figure 1c). The objective lens is focused in the z-direction to obtain a Raman signal, and the movement of the objective lens in the x (or y) direction is not considered. Therefore, the movement of the lens in the x (or y) direction is fixed in most Raman spectrometers. This study uses a simpler fabrication process of an OPU-based Raman spectrometer and modifies the Raman signal using the OPU tracking mode.

## 2 Experimental

### 2.1 Disassembly, replacement, and matching of the optical path

To fabricate the Raman probe, the red laser diode (LD) and circuit connections in the OPU (KSS–213C) were first removed and disconnected (Figure S1). The OPU base was attached to a three-dimensional (3D) printed OPU support (all 3D printed parts, including sample holders, are described in the Supplementary Information). Then, a 520-nm LD (PLT5 520B, 80 mW, OSRAM) with 3D-printed housing and a collimator (SHHO, M5 focal length: 6 mm) was attached to the OPU base. Before attaching the LD module, the diode was soldered and the collimated beam output was verified (Figure S1b). A beam splitter was used to align the optical path of the laser beam while mounting the laser module before replacing it (Figure S1c). Then, the beam splitter was replaced with a Ag dot-type reflective mirror (the fabrication method is described later in this section). When the beam splitter was replaced with a Ag mirror, the same method for attaching the laser module (Figure S1c) was used to match the optical path, thus allowing the beam to be placed at the center of the object lens. The 3D-printed collimator adapter was then attached to the OPU base, with the adapter hole matching the Ag dot mirror (Figure S1d). Two pins (14(−) and 15(+)) were soldered on the control board to use the OPU tracking mode to use the OPU tracking mode. The circuit was then activated by removing the solder from the board (Figure S1f). The adapter was fitted with a Raman-edge filter (Ø12.5 mm, 537.3 nm, Edmund Optics) and a collimator (10-mm aperture collimator VIS-NIR, Edmund Optics). Finally, an optical fiber (SMA-type, 400-μm core diameter) was used to connect a spectrometer (R spectrometer, Thunder Optics) to the collimator. The power sources used were a function generator (Owon, AG051F) and a power amplifier (DPA-2698, Juntek). All Raman spectra were obtained at a fixed voltage (6.6-V, 1-kHz square pulse), 30% duty ratio, and 8-s integration time. The designs of the 3D printed parts are shown in the Supplementary Information (Figure S2 to S6).

### 2.2 Fabrication of silver dot mirror

An Ag dot mirror was selected over a regular beam splitter because the latter is a half mirror; thus, only 50% of the incident light is reflected or transmitted. Therefore, the intensity of light decreases by 50% every time it passes through the beam splitter, making it difficult to observe a weak Raman signal. Similar issues have been reported in previous studies [23]. Ag dot reflection mirror was formed by Ag mirror reactions during a lift-off process (Figure S7). An electronic cutting tool was used to prepare engraved circle patterns on commercial waterproof label paper (Silhouette Cameo). A pattern-bonded glass substrate was placed inside the reaction solution to enable the adhesion of Ag to the mirror. Ag mirror reactions occurred for 1 min after ∼5 min of surface cleaning in a basic solution. The Ag-dot-coated glass was then cut into 7 and 5 mm and used as reflection mirrors.

## 3 Results and discussion

**Figure 2:**
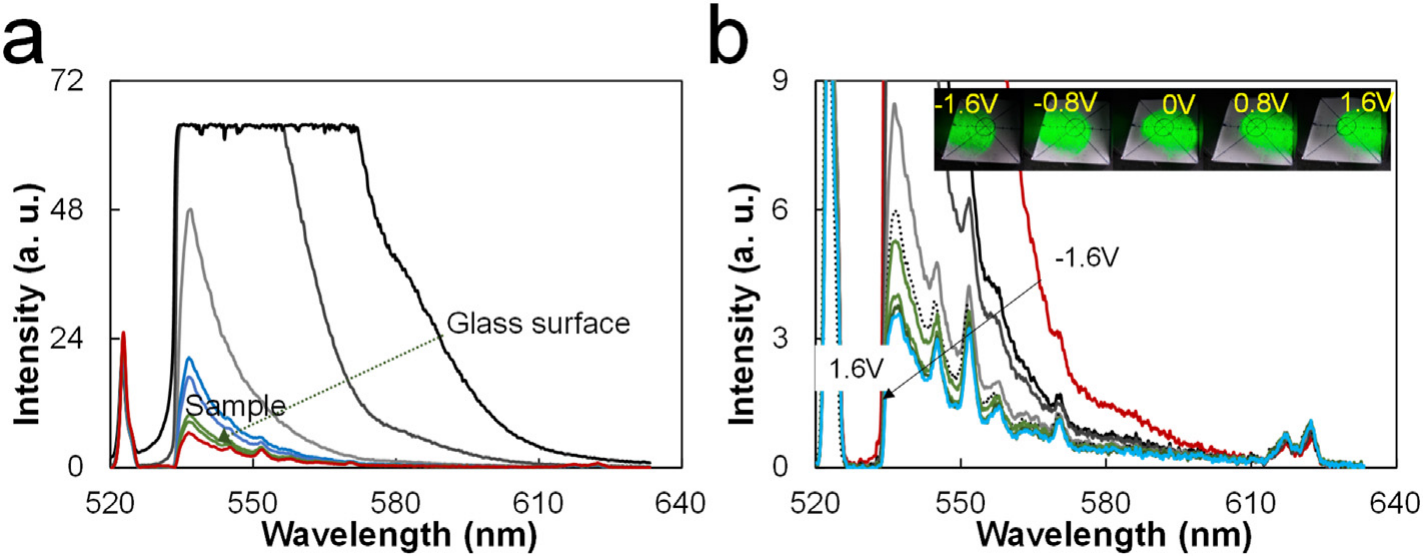
OPU-Raman signal focus: (a) Raman spectra as a function of the z distance between the objective lens and the sample (the large reflection of the fluorescence on the surface of the glass vial is first found); (b) Raman spectra obtained using the OPU tracking mode. The inset shows the bottom of an OPU-Raman probe box, with the marked paper showing the reflected laser beam. The spectra were recorded against toluene with an 8-s integration time, 30% duty ratio and 6.6-V (1-kHz) squared pulse.

The object lens of an OPU can be moved toward the z and x (or y) directions using electromagnetic coils, thus allowing the laser beam to be focused on the sample. Here, a z stage rather than the z-focusing mode of the OPU was used to simplify the analysis. To focus the beam on the sample, we must locate a point on the surface of the vial (the sample vessel that provides a large fluorescence signal, Figure 2a). Then, by increasing the height of the z stage, the beam will be focused on the sample placed within the vial, thus allowing Raman signals of the sample. The z-direction is used to focus the laser beam in all the cases of a typical Raman system. The x (or y) direction and the tracking mode of the OPU is a unique model that cannot be replaced by another x (or y) stage because the lens moves along the x (or y) axis. After z focusing, the objective lens is moved using the OPU tracking mode. Moving the lens in the x (or y) direction reduces the background signal intensity and increases the Raman signals (Figure 2b). Because the OPU exhibits strong Rayleigh scattering and fluorescence (discussed later), the OPU tracking mode can be a critical feature for OPU-based Raman probes. Moreover, using the tracking mode of an OPU can compensate for optical mismatches caused by the replacement of original parts or can be used to purposefully mismatch the optical path to reduce fluorescence.

**Figure 3:**
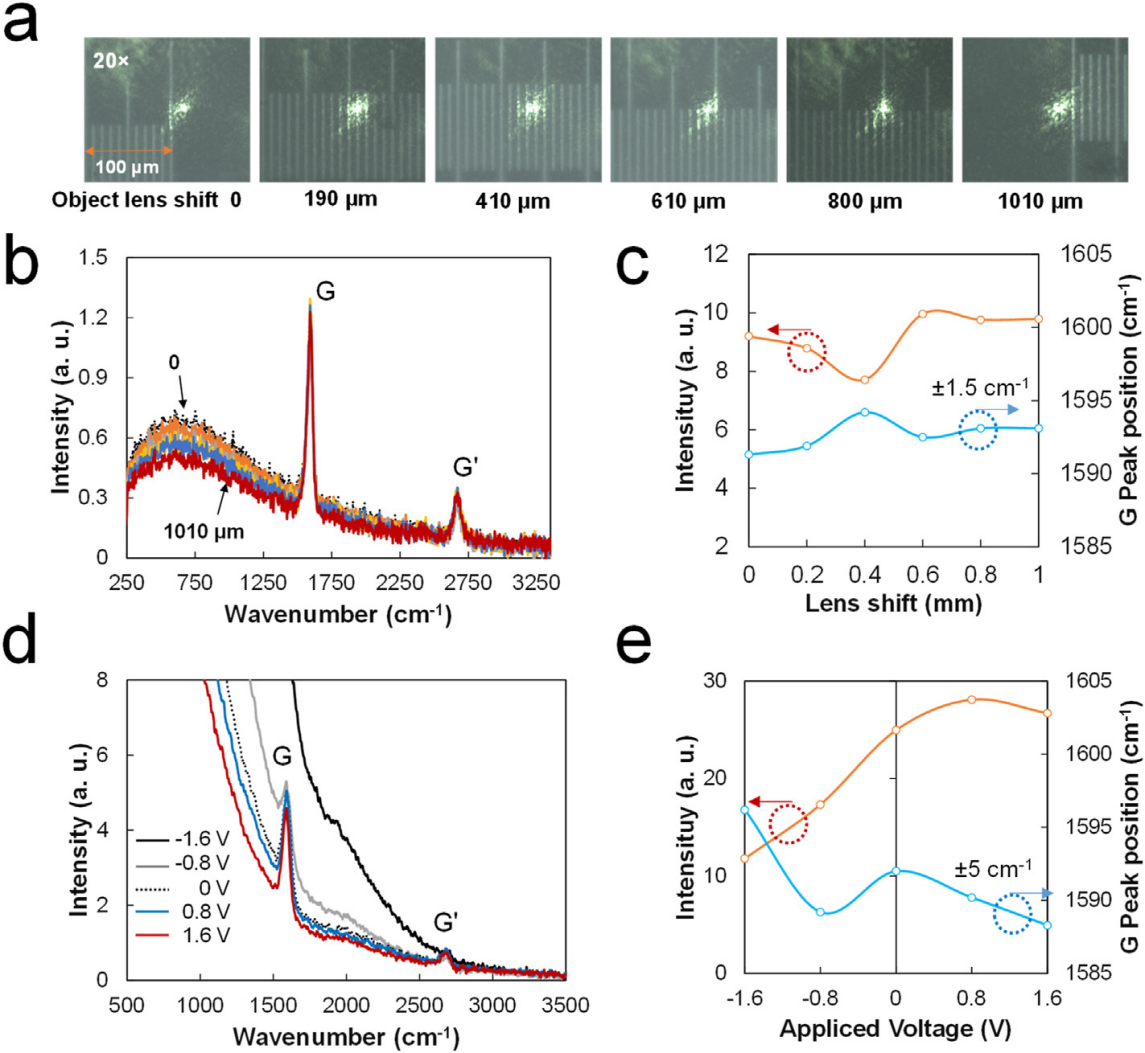
Raman spectra comparison by objective lens horizontal position: (a) microscopic image of laser spot against lens position in the commercial micro-Raman system, (b) raw Raman spectra of SWCNT from (a), (c) intensity and G peak position of each spectrum of (b), (d) OPU-Raman spectra of SWCNT depending on the applied voltage in the tracking mode, and (e) intensity and G peak position of each spectrum of (d).

**Figure 4:**
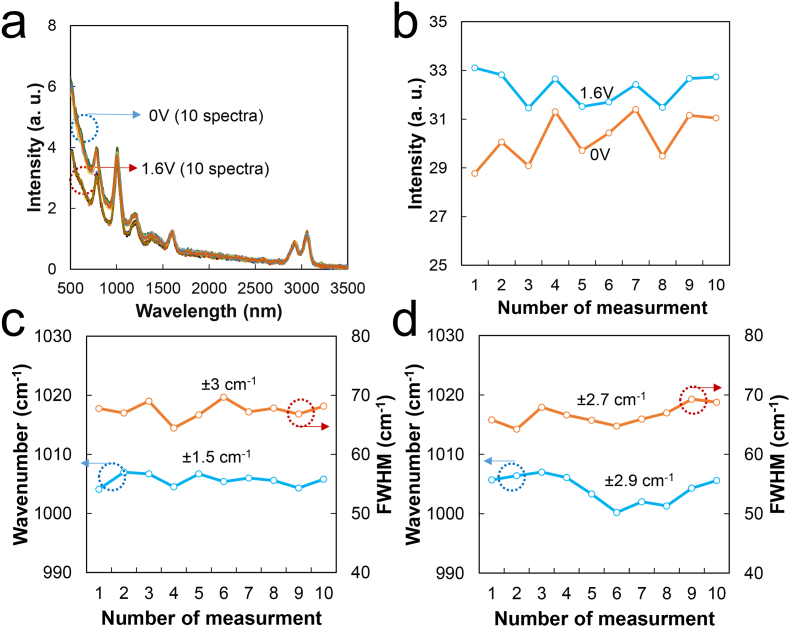
OPU Raman probe reproducibility test; (a) 10 normal and lens-shifted Raman spectra of toluene, (b) intensity variation of (a) at ∼1003 cm^−1^, (c) variation of the peak position and FWHM (lens-shifted Raman spectra), and (d) variation in the peak position and FWHM (normal Raman spectra).

The author investigated whether the Raman spectrum of a typical Raman spectrometer could provide a similar change as per lens movement in the x-direction. Because a micro-Raman system uses a microscope with a movable circular head to change magnification, the objective lens can be slightly moved in a horizontal direction without defocusing (Figure 3a). The Raman spectra of spin-coated single-walled carbon nanotubes (SWCNTs, OCSiAl, COAT_E, 1.6 nm diameter, 0.2 wt.% dispersion in H_2_O) placed on a scaler were obtained without additional focusing by horizontally moving the objective lens of a micro-Raman spectrometer (UniRam, UniNanoTech) by ∼0.2 mm from 0 to 1 mm. The Raman spectrum of the sample shows that the fluorescence intensity slightly decreases with increasing distance in the raw spectra shown in Figure 3b. The G peak (in-plane C=C bond stretching mode) intensity slightly decreased but showed similar G peak positions (Figure 3c). The tracking mode of the OPU-Raman probe was used to obtain SWCNT Raman spectra, which were compared to those obtained using the micro-Raman system. A 0.1-ml SWCNT mixture was dropped in a 2-ml vial and dried at 80 °C for 3 h. The vial was then placed upside down in the sample holder. The raw Raman spectrum, indicating the change of G peak intensity and background fluorescence with a voltage applied to the coil in the tracking mode, is shown in Figure 3d. The resulting Raman spectra show an increase in the G peak intensity with increase in voltage (Figure 3e). Moreover, the G position of the SWCNT sample differs by less than ±5 cm^−1^ against lens shift. The author investigated the OPU Raman probe reproducibility for the tracking mode. Figure 4a shows the lens shift (applied 1.6 V to tracking mode) and typical (0 V) Raman spectra obtained for toluene (10 spectra each taken after periodic laser power on/off). The tracking mode Raman (1.6 V) has the highest peak of 1003 cm^−1^, indicating that it has a higher intensity than that of the normal Raman mode of the OPU (Figure 4b). The peak position deviation was calculated to be around ±3 cm^−1^, and the deviation of the FWHM value was reported to be similar (Figures 4c and d).

**Figure 5:**
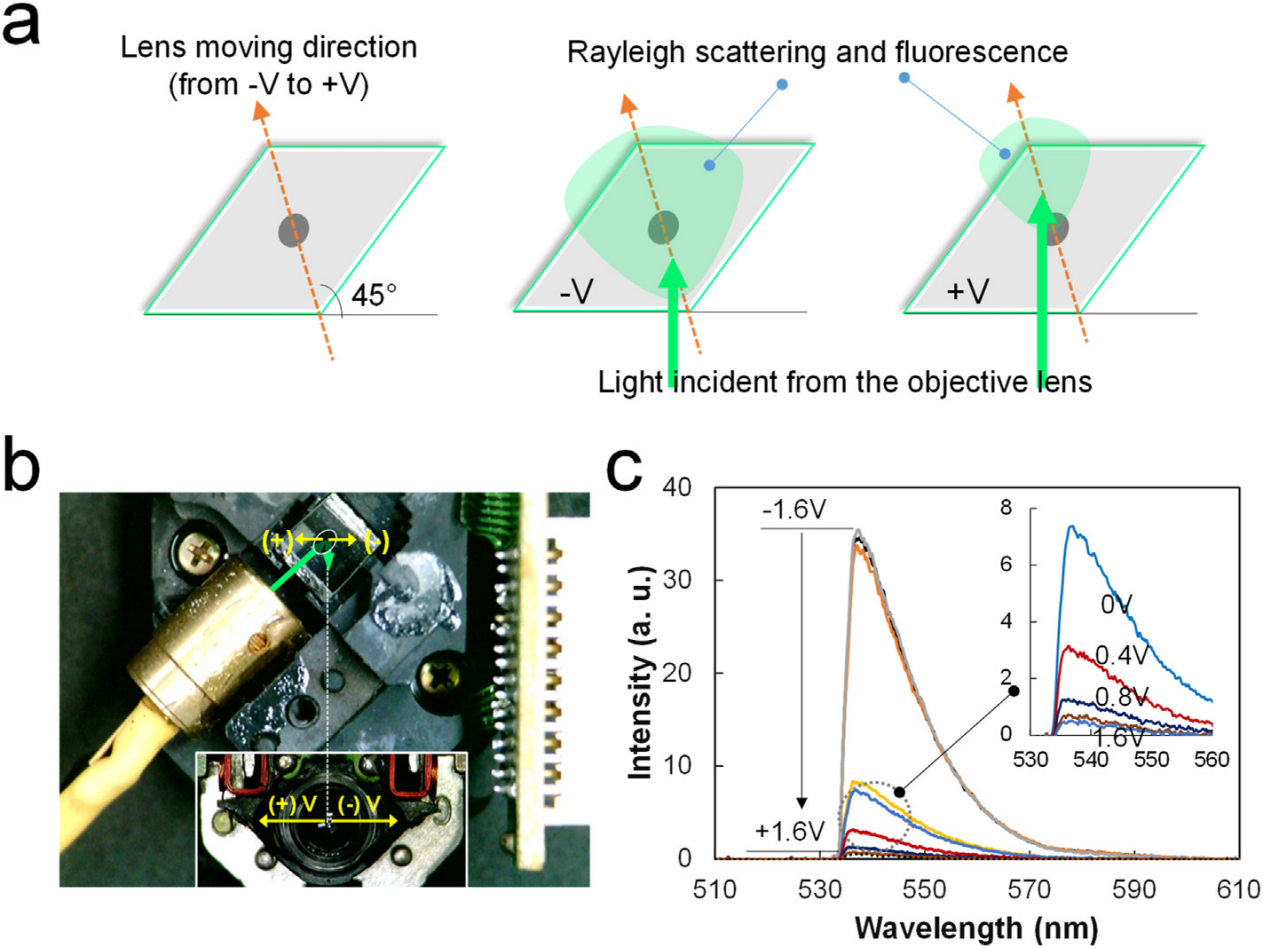
A dot mirror surface scattering model of collimated light from the objective lens; (a) light scattering diagram of the dot mirror surface, (b) a photo image showing the trajectory of the collimated light by the tracking mode, and (c) light scattering spectra (measured at 0.4% duty ratio, and 5.8-V (1-kHz) squared pulse) from a highly reflective Si wafer dependent on lens shift.

The scattering of the incident light on the surface of the dot mirror (Figure 5a) can explain the increase in Raman spectra and decrease in background signals. First, we can consider two optical passes of reflected light through the center and 0.8-mm positions in the x-direction from the center of the objective lens. The dot mirror not only reflects the laser but also blocks the passage of lights from the objective lens, including Raman signals. The scattering of fluorescence from this mirror surface becomes the background signal of Raman spectra. When a voltage is applied to the coils, the objective lens is moved toward the upper left (+V) or lower right (−V) direction of the Ag dot mirror (Figure 5b). The light scattering of the mirror surface is expected to be minimized in the upper left, and the part obscured by the dot mirror is slightly avoided, thus resulting in an increase in Raman signals. To confirm this effect, we measured the intensity of incident lights (acting as a background signal in the Raman spectrum) from the objective lens using a Si wafer with high reflectivity. A significant decrease in reflected light can be observed as the applied voltage to the tracking mode increases from −1.6 to +1.6 V (Figure 5c).

**Figure 6:**
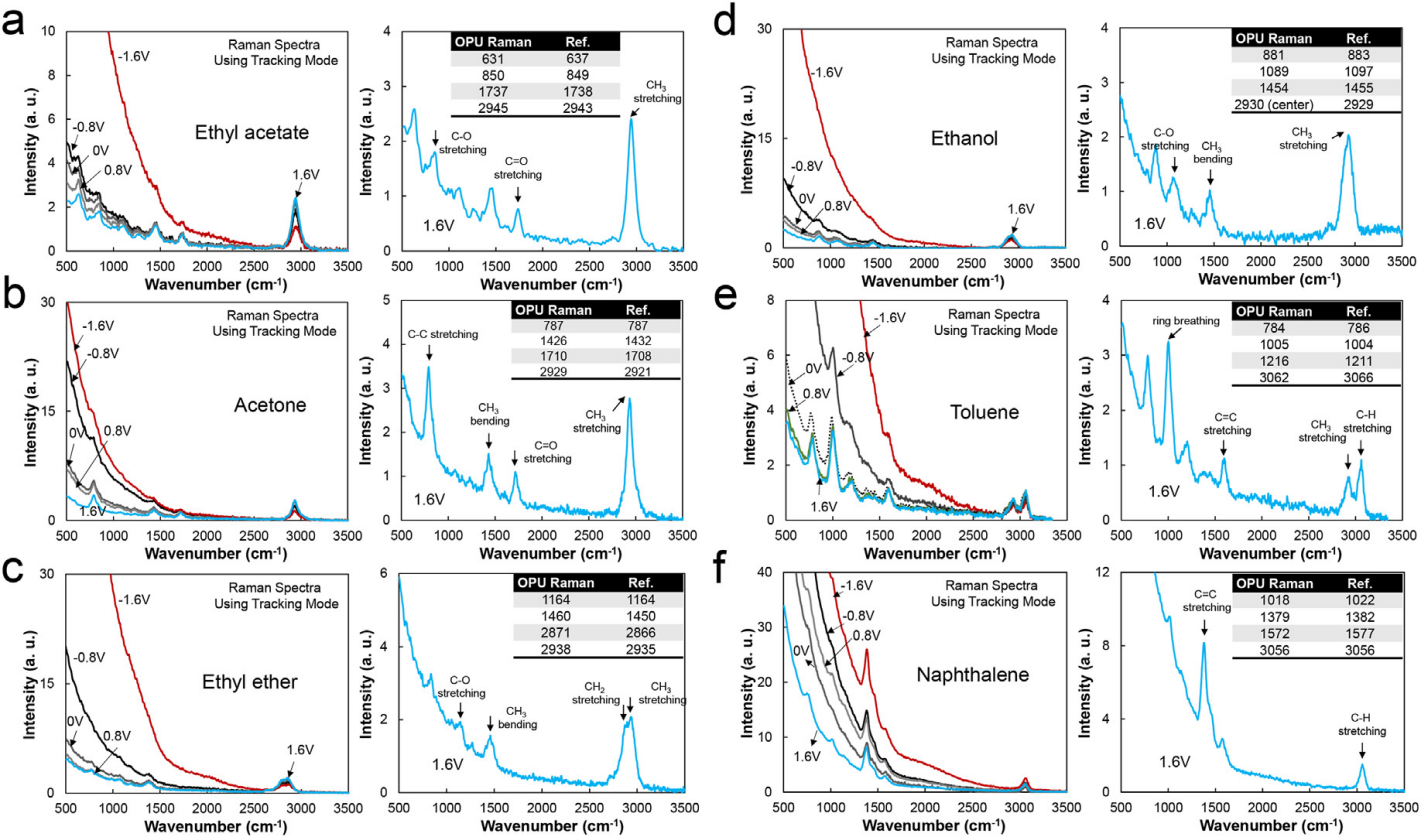
Raman spectra of liquid organic solvents (a) ethyl acetate, (b) acetone, (c) ethyl ether, (d) ethanol, (e) toluene, and (f) (solid) naphthalene. The right-side spectrum is a selected spectrum among left-side spectra obtained under the tracking mode. Raman spectra were recorded in OPU tracking mode with an 8-s integration time, 30% duty ratio, and 6.6-V (1-kHz) squared pulse. The inset table compares the OPU Raman peaks of each sample with the reported Raman peaks.

The Raman spectra of various organic solvents and a solid sample of naphthalene were obtained (Figure 6). All Raman spectra were recorded at an 8-s integration time, 30% duty ratio, and 6.6-V (1-kHz) square-pulsed power. The inset tables in Figure 6 compare the major peaks obtained with OPU-Raman and commercial Raman systems [25]. The Raman spectra in Figure 6 show that the tracking mode reduces the background signals and clarifies the Raman signals. The maximum position difference is ∼10 cm^−1^ for all samples, demonstrating considerable positional accuracy of spectra obtained with the OPU-Raman system. Though some sample peaks are not well resolved, multiple major characteristic peaks are observed. The carbonyl group is observed in the spectra of ethyl acetate and acetone (Figures 6a and b) at 1737 and 1710 cm^−1^, respectively. This result indicates that the carbonyl group in esters and ketones can be identified using OPU-Raman spectra. The Raman spectrum of ether (Figure 6c) shows two stretching peaks of –CH_2_– and –CH_3_ at 2871 and 2938 cm^−1^, respectively. A significant difference is observed between Raman and Fourier transform infrared (FT-IR) sample spectra. First, the Raman spectrum of ethyl acetate (Figure 6a) shows a strong –CH_3_ stretching mode at ∼2945 cm^−1^, whereas its FT-IR peak has low intensity compared with other vibrational modes. However, the spectrum of ethanol (Figure 6d) has extremely low intensities of –OH vibrational modes at ∼3400 cm^−1^ compared with that of the FT-IR spectrum. For toluene (Figure 6e), OPU Raman showed a difference in vibrational modes between sp^2^ C–H (3062 cm^−1^) and sp^3^ C–H. The OPU tracking mode could clarify the Raman signal for the solid naphthalene sample, but the signal intensity was slightly reduced because of the irregular surface shape of the sample (Figure 6f).

## 4 Conclusion

In summary, an OPU-based Raman spectrometer fabrication and signal tracking method was demonstrated. The completed OPU-Raman spectrometer has a maximum position difference of ∼10 cm^−1^ for all samples, indicating that the spectra obtained from this system have significant position accuracy. The OPU tracking mode of this system is a unique feature. Typical Raman systems use only the z-direction to focus and collect Raman signals, whereas the fabricated OPU-Raman system can move in the x (or y) direction (OPU tracking mode). The obtained spectra demonstrated enhanced Raman and reduced background signals because of the decrease in light scattering on the dot mirror surface caused by changing optical passes of reflected light and Raman signal blocking by the dot mirror. The obtained organic spectra show significant differences between Raman and FT-IR spectroscopic techniques. In particular, the different intensities of specific peaks of the –CH_3_ stretching mode for ethyl acetate and –OH vibrational modes in ethanol are observed using Raman and FT-IR spectroscopy. Based on these results, the OPU-Raman system is expected to be extensively built and used, even in educational and research environments where budgets are largely insufficient.

## Declarations

### Author contribution statement

Sung Il Ahn: Conceived and designed the experiments; Performed the experiments; Analyzed and interpreted the data; Contributed reagents, materials, analysis tools or data; Wrote the paper.

### Funding statement

This work was supported by Basic Science Research Program through the National Research Foundation (No. 2018R1A5A1025594 and No. 2019R1A2C1006771) by the Ministry of Science, ICT of Korea.

### Data availability statement

Data will be made available on request.

### Declaration of interests statement

The authors declare no conflict of interest.

### Additional information

Supplementary content related to this article has been published online at [URL].

## Appendix A Supplementary data

r-OPU-RAM-s-info-rev r-OPU-RAM-s-info-rev
