# Supplementary material for: A study on signal enhancement of a Raman probe using an optical pickup unit
Source: Heliyon. 2022 Sep 29;8(10):e10802. doi: 10.1016/j.heliyon.2022.e10802 (PMC9547214; doi:10.1016/j.heliyon.2022.e10802)
Supplement: r-OPU-RAM-s-info-rev [file mmc1.docx]

**A study on signal enhancement of a Raman probe using an optical pickup unit**

Sung Il Ahn

Department of Chemistry Education, Graduate Department of Chemical Materials, Institute for Plastic Information and Energy Materials, Pusan National University, Busandaehakro 63-2, Busan 46241, Republic of Korea

**1. Fabrication of OPU Raman-probe**

**2. 3D printed parts (3D CAD design)**

**3. Fabrication of Ag dot mirror**

**1. Fabrication process of the OPU Raman-probe**

Figure S1. Raman probe fabrication process and optical images of each process. (a) Removal of the red laser diode (LD) and sensor, disconnecting unnecessary circuit connections of the LD and sensor (the wire for the electromagnetic coil is not disconnected). (b) Collimation of the LD module. (c) Attachment of the laser module using the original beam splitter to maintain the OPU optical path (the paper in the images makes it easier to focus the laser module). (d) Attachment of a holder for the collimator and Raman filter. (e) Image of the finished OPU-Raman probe. (f) Soldering to activate the tracking mode and circuit.

**3. 3D printed parts (3D CAD design)**

**Figure S2**. (a) Adapter for a laser diode and collimator; (b) laser diode pin protector.

**Figure S3**. OPU base holder

**Figure S4**. Holder for Raman edge filter and collimator

**Figure S5**. (a) Liquid and (b) solid sampler holders

**Figure S6**. Light-block box

**3. Fabrication of Ag dot mirror**

**Figure S7**. Schematic of Ag dot mirror fabrication using a lift-off process.
